# Prevalence and incidence of primary autoimmune hemolytic anemia and cold agglutinin disease in the United States, 2016–2023

**DOI:** 10.1371/journal.pone.0323843

**Published:** 2025-06-26

**Authors:** Sylvie Bozzi, Siddhi Umarje, Kalyani Hawaldar, Jennifer Tyma, Brad Ward, Jill Schinkel, Barnabie Agatep, Zulkarnain Pulungan, Natalia Petruski-Ivleva

**Affiliations:** 1 Sanofi, Gentilly, France; 2 Sanofi, Cambridge, Massachusetts, United States of America; 3 Inovalon, Bowie, Maryland, United States of America; Karolinska Institutet, SWEDEN

## Abstract

**Background and aims:**

Autoimmune hemolytic anemia (AIHA) and cold agglutinin disease (CAD) are debilitating conditions characterized by chronic hemolysis and severe anemia. The existing epidemiological estimates in the United States (US) remain limited because of the rarity of AIHA and CAD. This retrospective study aims to update the epidemiology of AIHA and CAD in the US from 2016 to 2023 by separately analyzing administrative claims data from Optum’s de-identified Clinformatics^®^ Data Mart (Optum CDM), Inovalon’s Medical Outcomes Research for Effectiveness and Economics (MORE^2^) Registry, and Medicare Fee for Service (FFS).

**Methods:**

The study consisted of patients aged ≥18 years with 180 days of continuous enrollment in their health plan. The index date was the first observed claim with AIHA or CAD ICD-9/10 diagnosis codes. The 2016–2021 US Census data were used to standardize incidence and prevalence estimates by age and sex. The results were presented by the US states.

**Results:**

The AIHA incidence ranged from 1.4 to 6.6 per 100,000 persons across the databases. The CAD incidence ranged from 0.6 to 1.2 per 100,000 persons across the databases, with Medicare FFS estimates being the highest. Prevalence estimates also varied, with the AIHA 1-year prevalence ranging from 4.2 to 20.6 per 100,000 persons and CAD from 1.4 to 3.1 per 100,000 persons across the databases.

**Conclusion:**

This multi-database analysis provides the updated epidemiological estimates of AIHA and CAD in the US, showing a higher incidence and prevalence in females than males, with both conditions increasing with age. However, no clear geographic pattern emerged across the US.

## 1. Introduction

Autoimmune hemolytic anemia (AIHA) is an acquired disorder wherein autoantibodies target red blood cells, leading to decompensated hemolysis and anemia. It is a rare blood disorder with an estimated incidence of 0.8 to 3 cases per 100,000 persons per year and a prevalence of 17 per 100,000 persons across multiple countries [1,2]. AIHA has a complex etiology, ranging from idiopathic origins to associations with conditions such as lymphoproliferative disorders, systemic lupus erythematosus, Crohn’s disease, or infections, as well as certain medications [1,3–6].

Based on the isotype and thermal characteristics of autoantibodies, AIHA can be categorized into warm, cold (including cold agglutinin disease [CAD] and paroxysmal cold hemoglobinuria), and mixed subtypes [7]. CAD is a rare subtype of AIHA mediated by the activation of the classical complement pathway and characterized by chronic hemolysis, severe anemia, fatigue, weakness, dizziness, and circulatory problems such as acrocyanosis and Raynaud’s phenomenon [5,8].

Cold agglutinin disease accounts for approximately 15% to 20% of AIHA cases [9,10] and usually occurs in people over 50 years, most often in the seventh and eighth decades of life [11,12]. Patients with CAD are at increased risk of thromboembolism and early mortality [3,13]. CAD negatively impacts the quality of life, contributing to fatigue, depression, and anxiety [14–19].

Despite advances in the understanding of AIHA and CAD, there is still a significant knowledge gap regarding the epidemiology of these diseases. Although AIHA is recognized as a serious health condition, the available estimates of its incidence and prevalence remain limited and often outdated [1,3–6]. This is particularly true for CAD, which, despite its impact on older adults, is underrepresented in contemporary epidemiological studies [1,3–6]. An accurate understanding of the disease burden of AIHA and CAD, including their geographical distribution and associated risk factors, is essential for improving clinical management and guiding public health initiatives. The lack of comprehensive, updated epidemiological data has hindered the development of targeted interventions and accurate healthcare resource allocation.

Although various treatments for CAD, such as rituximab, eculizumab, and corticosteroids, have been investigated, their effectiveness remains variable [12,20]. No universally approved therapies existed for CAD until the recent approval of sutimlimab by the Food and Drug Administration (FDA) in 2022 [21,22]. Rituximab, a B cell-depleting therapy, showed partial responses in approximately 50% of patients with CAD; however, relapses can occur as early as 2 months and typically within 6 months of treatment [23–27]. Rituximab, in combination with cytotoxic agents such as fludarabine or bendamustine, showed higher response rates in patients with CAD, however, it caused serious adverse events, including severe neutropenia, hematologic toxicity, and infections [28,29].

Eculizumab, a complement C5 inhibitor, reduced the need for transfusion. However, eculizumab increased hemoglobin levels modestly (by approximately 0.8 g/dL), as it did not manage extravascular hemolysis [30].

In contrast, sutimlimab showed a more significant increase in the hemoglobin levels (≥1.5 g/dL) of patients with CAD in the CARDINAL [21] and CADENZA [22] trials.

Sutimlimab targets complement-mediated hemolysis. Phase 3 studies have shown that sutimlimab rapidly halts hemolysis, improves hemoglobin levels, and reduces fatigue in patients with CAD, regardless of any recent transfusion history [21,22]. Sutimlimab as an effective treatment option for CAD, offers significant benefits in managing the condition and improving patient quality of life. However, to fully capitalize on these advancements, it is essential to understand the prevalence and distribution of CAD in diverse populations. A prevalence study can provide crucial epidemiological data, helping to identify treatment gaps and make informed future therapeutic strategies. Due to the rarity of AIHA and CAD, there has been inadequate epidemiological data in the United States (US).

This study aims to address these critical knowledge gaps by providing the updated incidence and prevalence estimates of AIHA and CAD in the US. By utilizing administrative claims data from commercial insurance, Medicare Advantage, Medicare Fee-for-Service (FFS), and managed Medicaid plans, this research offers a comprehensive view of the current burden of AIHA and CAD, which is essential for improving clinical care and informing public health planning.

## 2. Methods

### 2.1. Study design

**Fig 1:**
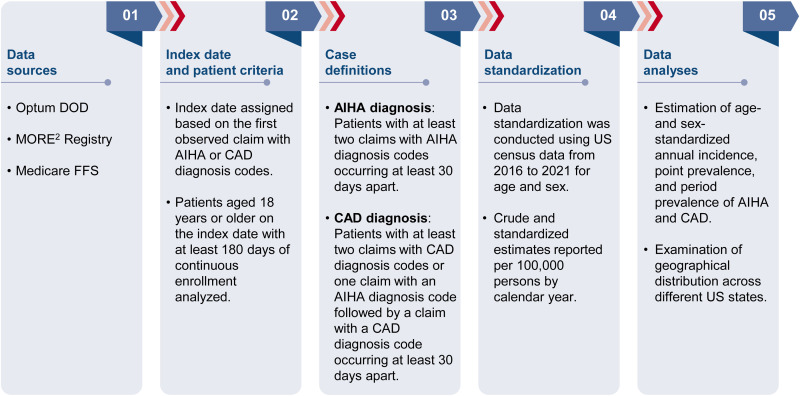
Study design flow chart. AIHA, autoimmune hemolytic anemia; CAD, cold agglutinin disease; FFS, Fee for Service; MORE^2^, Medical Outcomes Research for Effectiveness and Economics; Optum CDM, Optum de-identified Clinformatics^®^ Data Mart; US, United States.

This retrospective observational study estimated the age- and sex-standardized annual incidence, point prevalence, and period prevalence of AIHA and CAD in the adult US population from 2016 to 2023. The study also examined the geographical distribution of AIHA and CAD across the US states (Fig 1). We have adhered to the RECORD (REporting of studies Conducted using Observational Routinely-collected Data) checklist in the preparation of this manuscript. The RECORD checklist is provided in the supplementary materials (S1 File) [31]. Ethical approval was not required for this study. Consent to participate and consent to publish are not applicable.

### 2.2. Data sources

This analysis used administrative closed claims data from three sources: Optum’s de-identified Clinformatics^®^ Data Mart (Optum CDM), Inovalon’s Medical Outcomes Research for Effectiveness and Economics (MORE^2^) Registry, and 100% Medicare FFS.

Optum CDM contains the de-identified administrative health insurance claims data of over 90 million patients from May 01, 2000, to March 31, 2023. The database offers a comprehensive single-payer view, covering demographics such as gender, age, dates of eligibility, and plan type. It also includes diagnoses coded as per the International Classification of Diseases, Ninth or Tenth Revision (ICD-9 or ICD-10), procedures performed during outpatient visits or inpatient stays, and outpatient prescription records.

MORE^2^ Registry contains the de-identified medical and pharmacy claims data from January 01, 2000, to December 31, 2022. This real-world closed claims database is sourced from over 150 health plans. This database contains data gathered from over 1 million physicians, 594,000 clinical facilities, 342 million unique patients, and more than 65 billion medical events, offering a comprehensive view and generalizability of findings to the general US population. The claims data in the dataset were longitudinally matched and covered all major US payer lines of business, including commercial (31% of the market), Medicare Advantage (25% of the market), and managed Medicaid (89% of the market). All 50 US states were represented in this database, which could help update the incidence and prevalence estimates of AIHA and CAD across various geographical regions.

Medicare FFS contains the de-identified medical encounters data from January 01, 2015, to December 31, 2021. It includes all Part A/B medical encounters for beneficiaries, including hospital claims, emergency department visits, skilled nursing facility stays, hospital outpatient services/ambulatory surgical center services, physician office visits, home health services/durable medical equipment, and hospital care. Individuals qualify for Medicare FFS upon reaching the age of 65 years. However, younger patients with disabilities, end-stage renal disease, or amyotrophic lateral sclerosis are also eligible. As of 2021, 86.2% of Medicare FFS beneficiaries were 65 years of age or older.

### 2.3. Index date and patient criteria

The index date was the first observed claim with an AIHA or CAD ICD-9/10 diagnosis code (see the complete list in S2 File). Patients aged ≥18 years on the index date with at least 180 days of continuous enrollment (with a 30-day allowable gap) in their health plan before the index date were included. Patients with diagnosis codes for the secondary causes of AIHA or CAD, such as lymphoma, mucosa-associated lymphoid tissue lymphoma, chronic lymphoid leukemia, Waldenstrom’s macroglobulinemia, myeloma, or mycoplasma pneumonia, were excluded from the study. These conditions were evaluated at any point prior to and including the index date. Additionally, patients were excluded if they had diagnosis codes for mycoplasma, cytomegalovirus, or Epstein-Barr virus within 2 weeks prior to and including the index date [32]. The incidence of events was assessed from January 01 to December 31 of each calendar year. The denominator included the entire look-back period to identify prevalent cases and January 01 to December 31 of each calendar year to identify the eligible/at-risk population.

### 2.4. Case definitions

AIHA diagnosis was defined as patients having at least two claims with ICD-9/10 diagnosis codes for AIHA (283.0, D59.1, D59.10, D59.11, D59.12, D59.13, and D59.19), occurring at least 30 days apart between January 01, 2016 and March 31, 2023 for Optum CDM; December 31, 2022 for MORE^2^; and December 31, 2021 for Medicare FFS. CAD diagnosis was defined as patients having at least two claims with an ICD-10 diagnosis code for CAD (D59.12) or at least one claim with a diagnosis code for AIHA (283.0, D59.1, D59.10, D59.11, D59.12, D59.13, and D59.19), followed by at least one claim with a diagnosis code for CAD (D59.12), occurring at least 30 days apart.

### 2.5. Data standardization

The US Census data from 2016 to 2021 were used to standardize the incidence and prevalence estimates by age and sex. Due to the lack of US Census data for 2022 and 2023, estimates for these years were standardized using the 2021 data. Crude and standardized estimates were reported per 100,000 persons for each calendar year. The standardization method used was direct standardization, performed in Microsoft Excel, with data extraction conducted via Structured Query Language (SQL). Crude estimates were calculated as number of cases divided by eligible population size in the database, stratified by age group and sex. The crude estimate per stratum was multiplied by the corresponding US population size for that age and sex group based on US Census estimates, resulting in an estimated number of cases for each stratum in the US population. Finally, the estimated cases across all strata were summed, and the total estimated number of cases was divided by the total US population size to yield the population-standardized estimate.

### 2.6. Statistical analysis

The point prevalence of AIHA and CAD was defined as the prevalence of the condition at the start of each calendar year (i.e., January 01) among patients meeting the inclusion and exclusion criteria in the database. Point prevalence measured the condition’s prevalence in all prior available data within the specified study period. Period prevalence was defined as the proportion of patients meeting the disease criteria during each calendar year (January 01 to December 31). Incidence was defined as the total number of new cases in the given calendar year among patients with no prior evidence of AIHA in all data prior to the calendar year of interest. For CAD, estimates were presented from 2021 to 2023 due to the recent implementation of the CAD-specific ICD-10 code (active as of October 01, 2020). All the results were stratified by sex (female/male) and age group (in years; 18–24, 25–34, 35–44, 45–54, 55–64, 65–74, 75–84, and ≥85) to allow for standardization. No values were imputed. Heat maps were generated using R Statistical Software (v4.3.2; R Core Team 2023) packages usmap (v0.7.1; Di Lorenzo 2024) and ggplot2 (v3.5.2; Wickham 2016) to display the prevalence of AIHA and CAD across all 50 US states. The time range for incidence and prevalence was from January 01 to December 31 for each calendar year, and point prevalence was from January 01 and all time prior.

## 3. Results

### 3.1. Incidence and prevalence of AIHA

**Fig 2:**
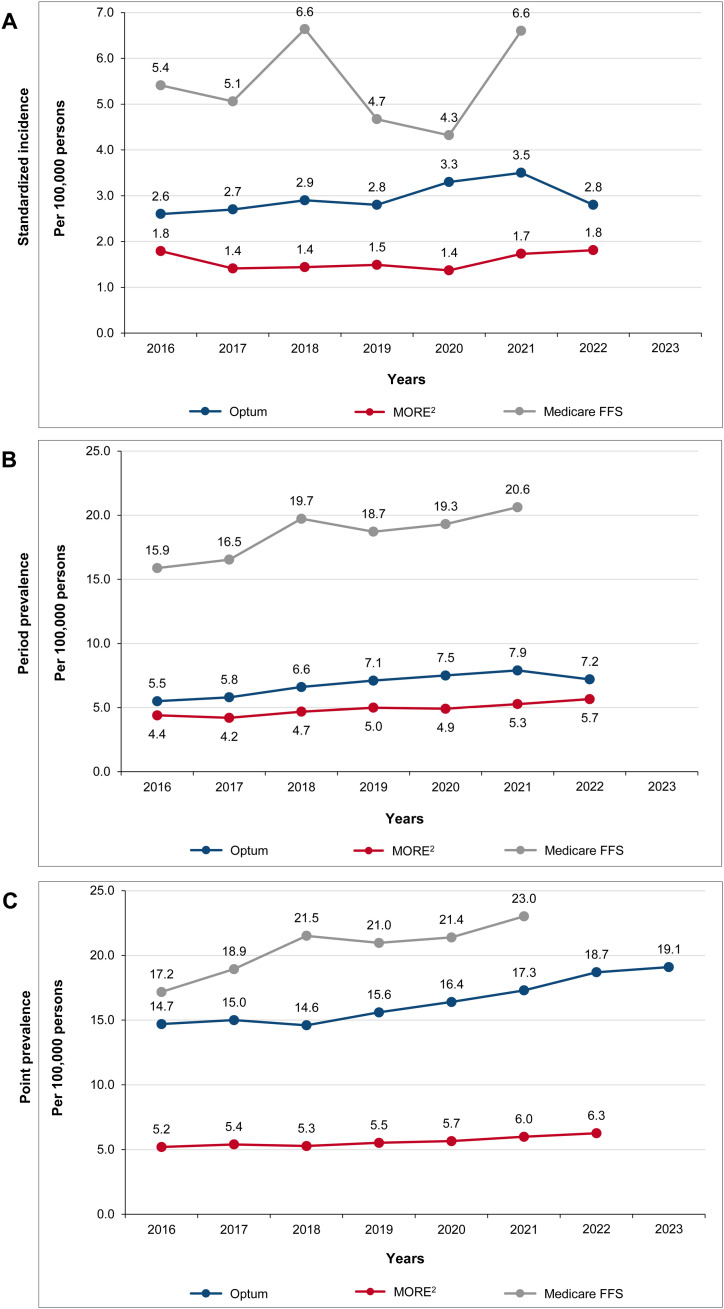
(A) Standardized incidence, (B) period prevalence, and (C) point prevalence of AIHA per 100,000 persons among adults aged ≥18 years in the United States, 2016 to 2023: Optum CDM, MORE^2^ Registry, and Medicare FFS. AIHA, autoimmune hemolytic anemia; CAD, cold agglutinin disease; FFS, Fee for Service; MORE^2^, Medical Outcomes Research for Effectiveness and Economics; Optum CDM, Optum de-identified Clinformatics^®^ Data Mart. Note: The incidence and prevalence numbers for CAD were relatively lower; hence, these numbers were not shown on the graphs.

The age- and sex-standardized incidence of AIHA in adults from 2016 to 2022 ranged from 2.6 to 3.5 per 100,000 persons in Optum CDM, 1.4 to 1.8 per 100,000 persons in MORE^2^ Registry, and 4.3 to 6.6 per 100,000 persons in Medicare FFS (Fig 2A). The 1-year prevalence of AIHA across 2016–2022 ranged from 5.5 to 7.9 per 100,000 persons in Optum CDM, 4.2 to 5.7 per 100,000 persons in MORE^2^ Registry, and 15.9 to 20.6 per 100,000 persons in Medicare FFS (Fig 2B). The point prevalence of AIHA across 2016–2023 ranged from 14.6 to 19.1 per 100,000 persons in Optum CDM, 5.2 to 6.3 per 100,000 persons in MORE^2^ Registry, and 17.2 to 23.0 per 100,000 persons in Medicare FFS (Fig 2C). Comparative estimates for both crude and standardized AIHA attributes were detailed in S2 File.

### 3.2. Incidence and prevalence of CAD

The incidence of CAD was 0.7 per 100,000 persons in Optum CDM (2022), 0.6 per 100,000 persons in MORE^2^ Registry (2022), and 1.2 per 100,000 persons in Medicare FFS (2021). The 1-year prevalence of CAD per 100,000 persons was 1.8 in Optum CDM (2022), 1.4 in MORE^2^ Registry (2022), and 3.1 in Medicare FFS (2021). The point prevalence of CAD was 3.1 per 100,000 persons in Optum CDM (2023), 1.6 per 100,000 persons in MORE^2^ Registry (2022), and 3.3 per 100,000 persons in Medicare FFS (2021). Comparative estimates for both crude and standardized CAD attributes were detailed in S3 File.

### 3.3. Point prevalence per 100,000 of AIHA cases across all 50 US states (non-standardized/crude estimates)

**Fig 3:**
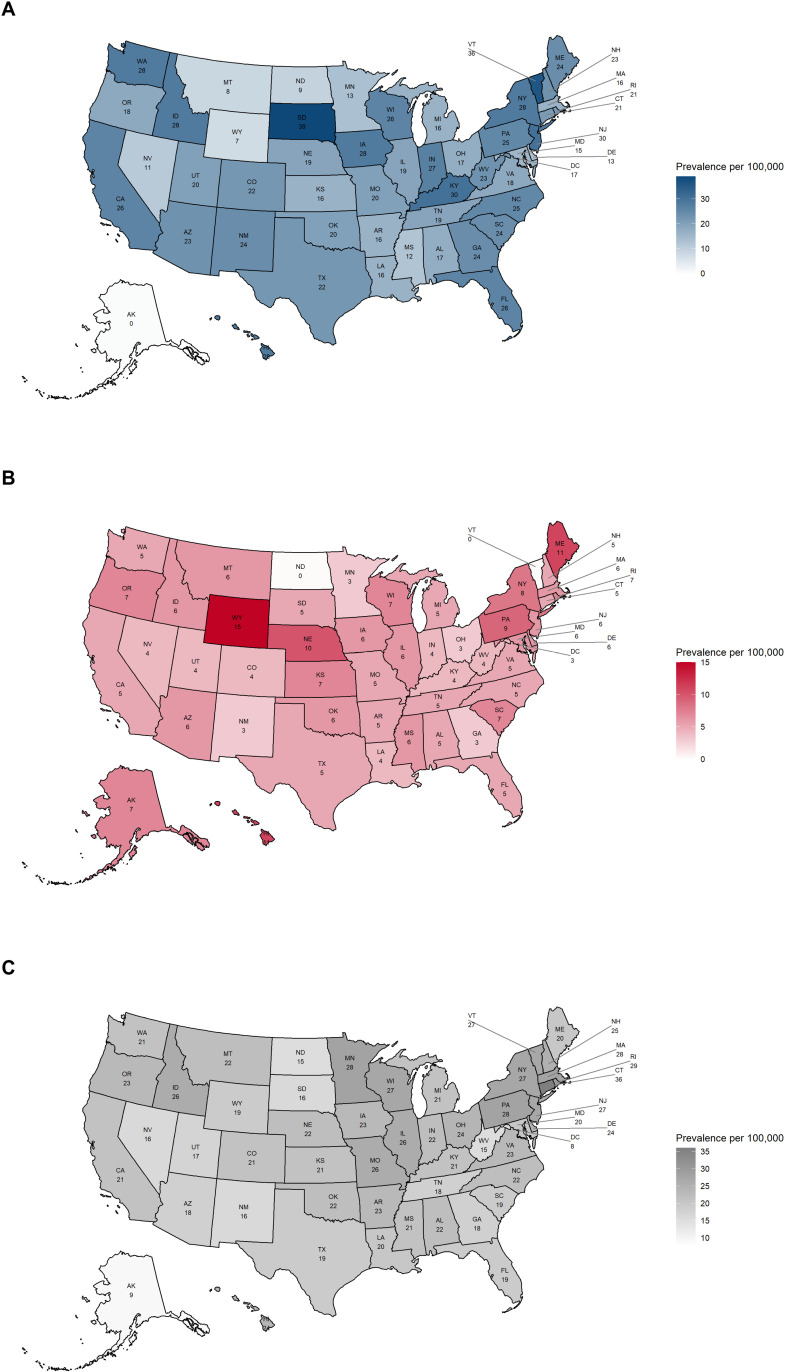
Point prevalence per 100,000 of AIHA among patients aged ≥18 years across the US states. (A) Optum CDM (2023), (B) MORE^2^ Registry (2022), and (C) Medicare FFS (2021). AIHA, autoimmune hemolytic anemia; FFS, Fee for Service; MORE^2^, Medical Outcomes Research for Effectiveness and Economics; Optum CDM, Optum de-identified Clinformatics^®^ Data Mart; US United States. AK, Alaska; AL, Alabama; AR, Arkansas; AZ, Arizona; CA, California; CO, Colorado; CT, Connecticut; DC, District of Columbia; DE, Delaware; FL, Florida; GA, Georgia; HI, Hawaii; IA, Iowa; ID, Idaho; IL, Illinois; IN, Indiana; KS, Kansas; KY, Kentucky; LA, Louisiana; MA, Massachusetts; MD, Maryland; ME, Maine; MI, Michigan; MN, Minnesota; MO, Missouri; MS, Mississippi; MT, Montana; NC, North Carolina; ND, North Dakota; NE, Nebraska; NH, New Hampshire; NJ, New Jersey; NM, New Mexico; NV, Nevada; NY, New York; OH, Ohio; OK, Oklahoma; OR, Oregon; PA, Pennsylvania; RI, Rhode Island; SC, South Carolina; SD, South Dakota; TN, Tennessee; TX, Texas; UT, Utah; VA, Virginia; VT, Vermont; WA, Washington; WI, Wisconsin; WV, West Virginia; WY, Wyoming.

In 2023, the top five US states with the highest point prevalence of AIHA (per 100,000) in Optum CDM were South Dakota (39), Vermont (36), New Jersey (30), Kentucky (30), and New York (28). Conversely, the lowest prevalence was recorded in Wyoming (7), Montana (8), North Dakota (9), Nevada (11), and Mississippi (12) (Fig 3A). The top five US states with the highest point prevalence of AIHA (per 100,000) in MORE^2^ Registry in 2022 were Wyoming (15), Maine (11), Hawaii (11), Nebraska (10), and Pennsylvania (10). The lowest prevalence was observed in Vermont (0), North Dakota (0), the District of Columbia (2.5), Minnesota (3), and Georgia (3) (Fig 3B). The top five US states with the highest point prevalence of AIHA (per 100,000) in Medicare FFS in 2021 were Connecticut (36), Rhode Island (28), Minnesota (28), Massachusetts (28), and Pennsylvania (27.5). The lowest prevalence was observed in Puerto Rico (0), the District of Colombia (8), Alaska (9), West Virginia (14.5), and North Dakota (15.5) (Fig 3C).

### 3.4. Point prevalence per 100,000 of CAD cases across all 50 US states (non-standardized/crude estimates)

**Fig 4:**
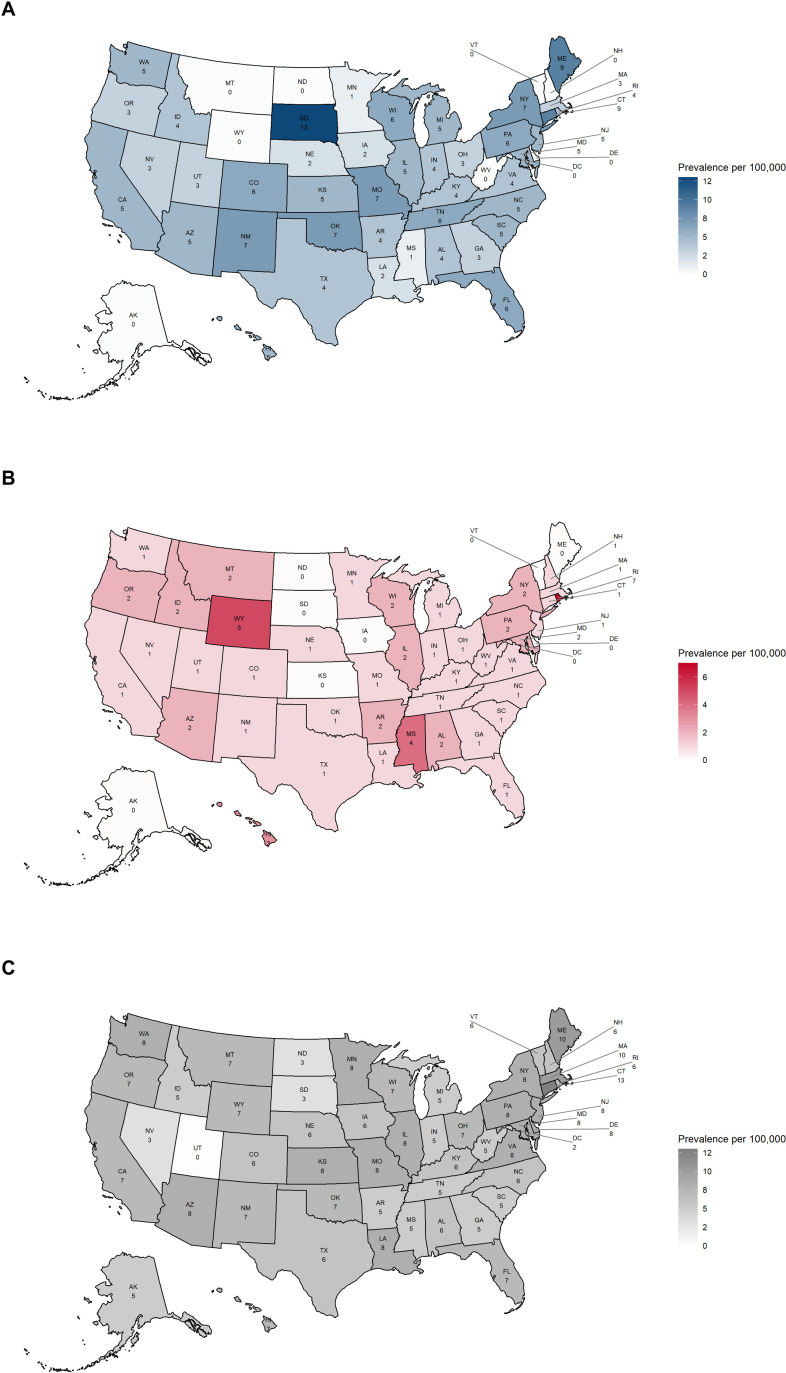
Point prevalence per 100,000 of CAD among patients aged ≥18 years across the US states. (A) Optum CDM (2023), (B) MORE^2^ Registry (2022), and (C) Medicare FFS (2021). CAD, cold agglutinin disease; FFS, Fee for Service; MORE^2^, Medical Outcomes Research for Effectiveness and Economics; Optum CDM, Optum de-identified Clinformatics^®^ Data Mart; US, United States. AK, Alaska; AL, Alabama; AR, Arkansas; AZ, Arizona; CA, California; CO, Colorado; CT, Connecticut; DC, District of Columbia; DE, Delaware; FL, Florida; GA, Georgia; HI, Hawaii; IA, Iowa; ID, Idaho; IL, Illinois; IN, Indiana; KS, Kansas; KY, Kentucky; LA, Louisiana; MA, Massachusetts; MD, Maryland; ME, Maine; MI, Michigan; MN, Minnesota; MO, Missouri; MS, Mississippi; MT, Montana; NC, North Carolina; ND, North Dakota; NE, Nebraska; NH, New Hampshire; NJ, New Jersey; NM, New Mexico; NV, Nevada; NY, New York; OH, Ohio; OK, Oklahoma; OR, Oregon; PA, Pennsylvania; RI, Rhode Island; SC, South Carolina; SD, South Dakota; TN, Tennessee; TX, Texas; UT, Utah; VA, Virginia; VT, Vermont; WA, Washington; WI, Wisconsin; WV, West Virginia; WY, Wyoming.

The top five US states with the highest point prevalence of CAD in Optum CDM in 2023 were South Dakota (13), Connecticut (9), Maine (9), New Mexico (7), and Oklahoma (7). The lowest prevalence was observed in Minnesota (1), Mississippi (1), Louisiana (2), Nebraska (2), and Iowa (2) (Fig 4A). The top five US states with the highest point prevalence of CAD in MORE^2^ Registry in 2022 were Rhode Island (6.5), Wyoming (5), Mississippi (4), Hawaii (3), and Pennsylvania (2.5). The lowest prevalence was observed in Vermont (0), South Dakota (0), Puerto Rico (0), North Dakota (0), and Maine (0) (Fig 4B). The top five US states with the highest point prevalence of CAD in Medicare FFS in 2021 were Connecticut (13), Massachusetts (10), Maine (9.5), Virginia (8), and Delaware (8). The lowest prevalence was observed in Utah (0), Puerto Rico (0), the District of Colombia (2), Nevada (3), and North Dakota (3) (Fig 4C). Notably, no geographic patterns were observed.

### 3.5. Distribution of AIHA and CAD by sex and age

**Fig 5:**
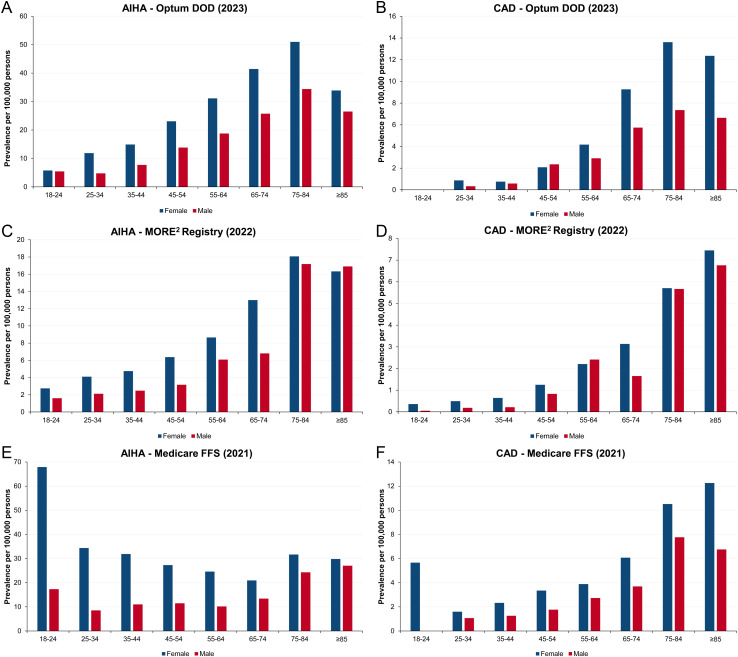
Point prevalence of AIHA and CAD by sex and age among adults aged ≥18 years in the United States: Optum CDM, MORE^2^ Registry, and Medicare FFS. AIHA, autoimmune hemolytic anemia; CAD, cold agglutinin disease; FFS, Fee for Service; MORE^2^, Medical Outcomes Research for Effectiveness and Economics; Optum CDM, Optum de-identified Clinformatics^®^ Data Mart.

The incidence and prevalence of AIHA and CAD were higher in females than males, increased with age, and were the highest among patients aged over 65 years for both conditions across the three databases (Optum CDM, MORE^2^ Registry, and Medicare FFS). For example, in 2023, the point prevalence of AIHA in the 65- to 74-years age group was 42 per 100,000 for females and 26 per 100,000 for males. In contrast, in the 25- to 34-years age group, the prevalence was notably lower at 12 per 100,000 for females and 5 per 100,000 for males (Fig 5). Similarly, in 2022, the point prevalence of CAD was about 6 per 100,000 persons in the 75- to 84-years age group, compared to less than 1 per 100,000 in the 25- to 44-years age group (Fig 5). However, these CAD proportions were slightly higher in females than in males.

## 4. Discussion

This study presents a multi-database analysis of the epidemiological estimates of AIHA and CAD among US adults from 2016 to 2023. The age- and sex-standardized incidence of AIHA ranged from 1.4 to 6.6 per 100,000 persons, while the point prevalence was 5.2 to 23.0 per 100,000 persons, and the 1-year prevalence was 4.2 to 20.6 per 100,000 persons across the three databases. In our study, for CAD, the point prevalence was 1.6 to 3.3 per 100,000 persons, the period prevalence was 1.4 to 3.1 and the incidence ranged from 0.6 to 1.2 per 100,000 persons across the three databases. Previously published literature reports the incidence of AIHA as approximately 2 per 100,000 persons and the prevalence as approximately 17 per 100,000 persons across North America and Western Europe [33].

Similarly, studies conducted across North America and Nordic countries report the incidence of CAD as approximately 0.3 per 100,000 persons and the prevalence as 1.6 per 100,000 persons [34]. Our study’s findings, while slightly higher for the CAD prevalence, are generally consistent with these previously published estimates and update the existing epidemiological estimates.

Although the standardized estimates varied across the three databases, the incidence and prevalence were similar when stratified by age group. This suggests that standardization did not fully account for underlying differences in the composition and representativeness of the overall US population across the three databases.

The prevalence and incidence were higher in females than males and peaked in those 65 years of age and older compared to younger age groups. Previous studies also observed that females were twice as likely as males to have AIHA and CAD [35,36]. The median onset of age for AIHA in males and females is approximately 65 years [33,35,36], a trend also observed in our study. The increased prevalence of AIHA among females may be attributed to hormonal influences, particularly estrogen, which can modulate immune system responses and increase susceptibility to autoimmune diseases. The elevated incidence of AIHA and CAD in older adults, particularly those over 75 years of age, can be explained by a combination of factors related to immunosenescence, the gradual decline of immune system function with age. This decline impairs body’s ability to regulate immune responses, thereby increasing susceptibility to autoimmune diseases. The high prevalence in the elderly population highlights the importance of considering aging a critical factor in the epidemiology of these conditions. Further investigation is required to fully understand the underlying mechanisms, including potential genetic and environmental factors that may contribute to these age- and sex-related differences [9,36–38].

The highest estimates were observed in Medicare FFS and the lowest in MORE^2^ Registry, likely due to differences in the age distribution of the populations. Medicare FFS primarily captures individuals over 65 years of age, while MORE^2^ Registry and Optum CDM include younger populations, primarily commercially insured patients, with MORE^2^ also covering those on Medicaid. Estimates from Optum CDM were similar to those from Medicare FFS, suggesting the elderly population is well represented in Optum CDM. The incidence and prevalence of AIHA and CAD among younger patients were higher in Medicare FFS, as young individuals with disabilities or severe illness are eligible for Medicare. Therefore, young patients with poor health are more likely to be captured in Medicare FFS than in other commercial insurance plans. Differences in estimates across databases arise from underlying differences in populations within these databases. Optum and MORE^2^ primarily include commercially insured younger populations, whereas Medicare consists largely of an elderly population. AIHA and CAD predominantly affect elderly individuals, they are more accurately captured in Medicare compared to the other two databases. However, younger populations with severe illnesses are also eligible for Medicare benefits, which may explain the higher estimates in Medicare databases as those with the coverage are more likely to have the disease.

The implications of these findings for public health and policy are significant. As the population ages, the disease burden is expected to increase, placing additional strain on healthcare systems. Healthcare providers need to be especially vigilant in diagnosing and managing these conditions in older adults. Public health policy should focus on improving early detection, particularly in high-risk populations, and support research into the mechanisms that drive the higher incidence among females and older individuals [9,36].

A key strength of our study is using three large US databases that represent the broader US population. Administrative claims databases are frequently used to study the epidemiology of conditions treated in insured patients. A fundamental limitation of this study is the limited patient observability, which restricts the length of the lookback period for assessing prevalence and incidence. In claims databases, only diagnosed cases can be measured, potentially overlooking undiagnosed cases. Variations in coding practices also introduce the possibility of misclassification of diagnoses, including AIHA and its subtypes. Additionally, the lack of a validated algorithm to identify AIHA or CAD cases from administrative claims may contribute to misclassification. To address this limitation, at least two claims 30 days apart with relevant diagnosis codes were required in the case definition. The lack of specific ICD codes for AIHA subtypes before October 01, 2020, limited the CAD analysis period to after 2021. Furthermore, Coding practices that use “unspecified” and “other” AIHA codes may lead to an underestimation of the percentage of CAD cases among AIHA patients. Additionally, the absence of specific diagnostic tests or medications for AIHA subtypes means that analysis relies solely on ICD-10 codes used for billing. To address this, the overall AIHA prevalence and incidence were estimated to update the epidemiology of specific AIHA subtypes. The non-Medicare population in MORE^2^ Registry was identified through a convenience sample. To account for this, US Census estimates were used to weight the unadjusted estimates and better reflect national projections. The geographical distribution of AIHA observed in the claims databases reflected the diagnosed prevalence or care sought across various US states. Thus, the observed prevalence in each state may represent not only residents but also those seeking care outside their state of residence.

## 5. Conclusion

The standardized prevalence and incidence of AIHA and CAD varied across the three databases. The MORE^2^ Registry showed the lowest estimates, reflecting younger Medicaid and commercially insured populations, while Medicare FFS showed the highest estimates, primarily capturing individuals aged ≥65 years. Trends were similar across age and sex in all three databases, with Medicare FFS being the most representative of the AIHA and CAD populations. AIHA and CAD affected females more than males and those above the age of 65 years. This study is the first comprehensive epidemiological analysis of AIHA and CAD in the US, using a robust approach that integrates three databases with diverse patient coverage.

## Supporting information

S1 File The RECORD checklist. (PNG)

S2 File Inclusion/exclusion criteria for AIHA and CAD analyses. (XLSX)

S3 File Crude and age/sex standardized prevalence and incidence of AIHA and CAD among 18 + years (per 100,000), United States, 2016–2023 – Optum CDM, More^2^ registry, and Medicare FFS. (DOCX)
