# Supplementary figures and images for: Prevalence and incidence of primary autoimmune hemolytic anemia and cold agglutinin disease in the United States, 2016–2023
Source: PLoS One. 2025 Jun 26;20(6):e0323843. doi: 10.1371/journal.pone.0323843 (PMC12200825; doi:10.1371/journal.pone.0323843)

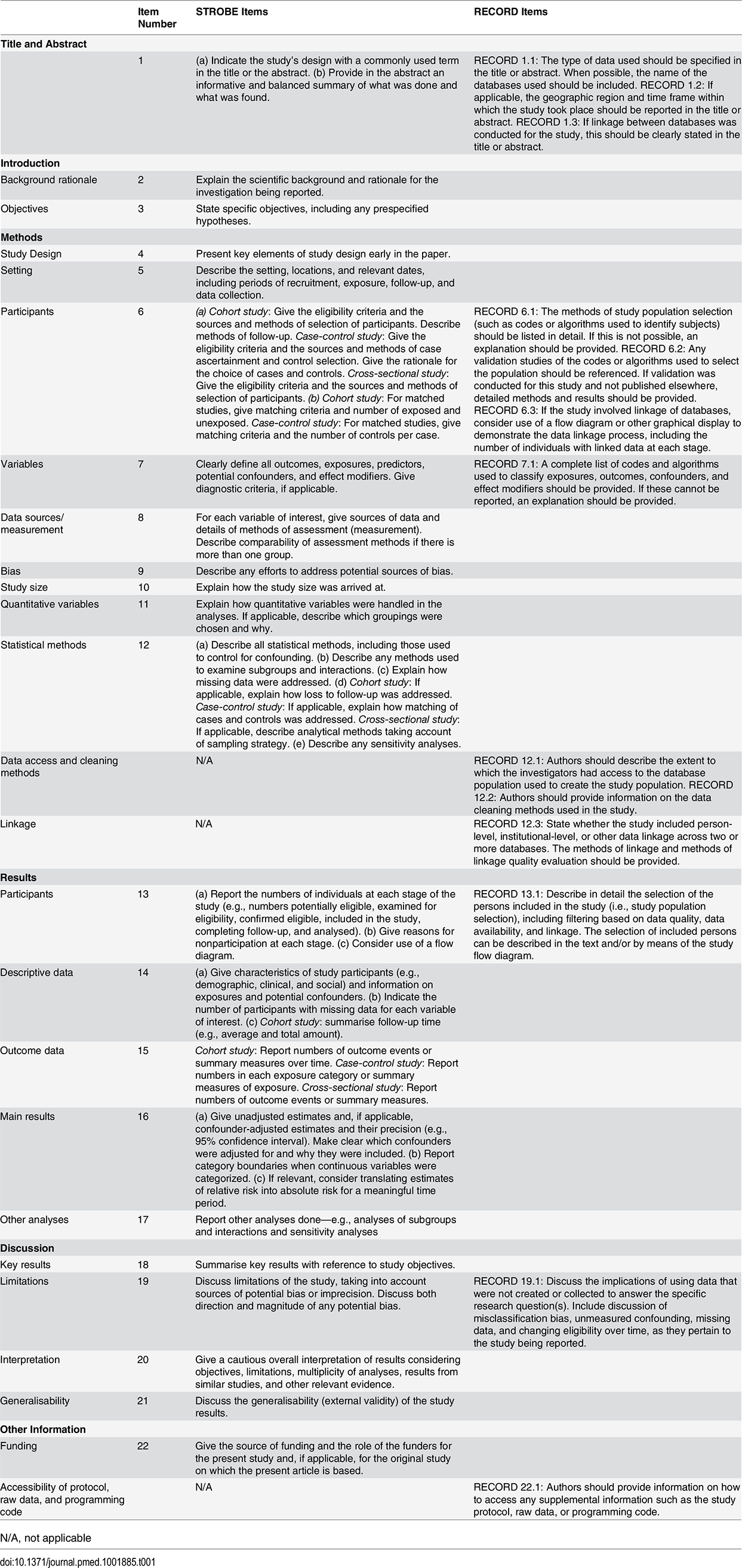

Supplement: S1 File — (PNG) [file pone.0323843.s001.png]
