# Supplementary material for: Prevalence and incidence of primary autoimmune hemolytic anemia and cold agglutinin disease in the United States, 2016–2023
Source: PLoS One. 2025 Jun 26;20(6):e0323843. doi: 10.1371/journal.pone.0323843 (PMC12200825; doi:10.1371/journal.pone.0323843)
Supplement: S3 File — (DOCX) [file pone.0323843.s003.docx]

**Table 1A: Crude and age/sex standardized prevalence and incidence of AIHA among adults 18+ years (per 100,000), United States, 2016 to 2023, Optum CDM**

| **Category** | **Estimates** | **2016** | **2017** | **2018** | **2019** | **2020** | **2021** | **2022** | **2023** |
| --- | --- | --- | --- | --- | --- | --- | --- | --- | --- |
| **Point Prevalence** | Crude | 17.5 | 18.3 | 18.3 | 20.1 | 21.6 | 23.0 | 25.4 | 26.2 |
|  | Standardized | 14.7 | 15.0 | 14.6 | 15.6 | 16.4 | 17.3 | 18.7 | 19.1 |
| **Period Prevalence** | Crude | 6.8 | 7.4 | 8.8 | 9.7 | 10.3 | 11.0 | 10.7 | 0.9 |
|  | Standardized | 5.5 | 5.8 | 6.6 | 7.1 | 7.5 | 7.9 | 7.2 | - |
| **Incidence** | Crude | 3.2 | 3.3 | 3.7 | 3.7 | 4.1 | 4.6 | 4.0 | 0.1 |
|  | Standardized | 2.6 | 2.7 | 2.9 | 2.8 | 3.3 | 3.5 | 2.8 | - |

AIHA, autoimmune hemolytic anemia; Optum CDM, Optum de-identified Clinformatics® Data Mart.

**Table 1B: Crude and age/sex standardized prevalence and incidence of CAD among adults 18+ years (per 100,000), United States, 2016 to 2023, Optum CDM**

| **Category** | **Estimates** | **2022** | **2023** |
| --- | --- | --- | --- |
| **Point Prevalence** | Crude | - | 5.4 |
|  | Standardized | - | 3.1 |
| **Period Prevalence** | Crude | 3.1 | - |
|  | Standardized | 1.8 | - |
| **Incidence** | Crude | 1.2 | - |
|  | Standardized | 0.7 | - |

CAD, cold agglutinin disease; Optum CDM, Optum de-identified Clinformatics® Data Mart.

**Table 2A: Crude and age/sex standardized prevalence and incidence of AIHA among adults 18+ years (per 100,000), United States, 2016 to 2022, MORE^2^ Registry**

| **Category** | **Estimates** | **2016** | **2017** | **2018** | **2019** | **2020** | **2021** | **2022** |
| --- | --- | --- | --- | --- | --- | --- | --- | --- |
| **Point Prevalence** | Crude | 4.5 | 4.6 | 4.7 | 4.9 | 5.2 | 5.3 | 5.3 |
|  | Standardized | 5.2 | 5.4 | 5.3 | 5.5 | 5.7 | 6.0 | 6.3 |
| **Period Prevalence** | Crude | 3.6 | 3.6 | 4.0 | 4.3 | 4.3 | 4.5 | 4.7 |
|  | Standardized | 4.4 | 4.2 | 4.7 | 5.0 | 4.9 | 5.3 | 5.7 |
| **Incidence** | Crude | 1.5 | 1.2 | 1.3 | 1.3 | 1.2 | 1.5 | 1.6 |
|  | Standardized | 1.8 | 1.4 | 1.4 | 1.5 | 1.4 | 1.7 | 1.8 |

AIHA, autoimmune hemolytic anemia; MORE^2^, Medical Outcomes Research for Effectiveness and Economics.

**Table 2B: Crude and age/sex standardized prevalence and incidence of CAD among adults 18+ years (per 100,000), United States, 2016 to 2022, MORE^2^ Registry**

| **Category** | **Estimates** | **2021** | **2022** |
| --- | --- | --- | --- |
| **Point Prevalence** | Crude | 1.2 | 1.2 |
|  | Standardized | - | 1.6 |
| **Period Prevalence** | Crude | 0.9 | 1.1 |
|  | Standardized | - | 1.4 |
| **Incidence** | Crude | 0.5 | 0.5 |
|  | Standardized | - | 0.6 |

CAD, cold agglutinin disease; MORE^2^, Medical Outcomes Research for Effectiveness and Economics.

**Table 3A: Crude and age/sex standardized prevalence and incidence of AIHA among adults 18+ years (per 100,000), United States, 2016 to 2021, Medicare FFS**

| **Category** | **Estimates** | **2016** | **2017** | **2018** | **2019** | **2020** | **2021** |
| --- | --- | --- | --- | --- | --- | --- | --- |
| **Point Prevalence** | Crude | 19.8 | 20.4 | 21.5 | 22.1 | 21.4 | 22.4 |
|  | Standardized | 17.2 | 18.9 | 21.5 | 21.0 | 21.4 | 23.0 |
| **Period Prevalence** | Crude | 18.3 | 18.8 | 19.9 | 20.5 | 19.9 | 21.0 |
|  | Standardized | 15.9 | 16.5 | 19.7 | 18.7 | 19.3 | 20.6 |
| **Incidence** | Crude | 5.7 | 5.2 | 5.3 | 5.0 | 4.6 | 5.8 |
|  | Standardized | 5.4 | 5.1 | 6.6 | 4.7 | 4.3 | 6.6 |

AIHA, autoimmune hemolytic anemia; FFS, Fee for Service.

**Table 3B: Crude and age/sex standardized prevalence and incidence of CAD among adults 18+ years (per 100,000), United States, 2016 to 2021, Medicare FFS**

| **Category** | **Estimates** | **2021** |
| --- | --- | --- |
| **Point Prevalence** | Crude | 6.8 |
|  | Standardized | 3.3 |
| **Period Prevalence** | Crude | 6.3 |
|  | Standardized | 3.1 |
| **Incidence** | Crude | 2.3 |
|  | Standardized | 1.2 |

CAD, cold agglutinin disease; FFS, Fee for Service.
